# The novel intubating laryngeal tube (iLTS-D) is comparable to the intubating laryngeal mask (Fastrach) – a prospective randomised manikin study

**DOI:** 10.1186/s13049-015-0126-y

**Published:** 2015-06-08

**Authors:** Thomas Ott, Matthias Fischer, Tobias Limbach, Irene Schmidtmann, Tim Piepho, Ruediger R. Noppens

**Affiliations:** Department of Anaesthesiology, University Medical Centre of the Johannes Gutenberg-University Mainz, Langenbeckstrasse 1, Mainz, 55131 Germany; Institute of Medical Biostatistics, Epidemiology, and Informatics, University Medical Centre of the Johannes Gutenberg University Mainz, Mainz, Germany

**Keywords:** Airway management, Intubation, Laryngeal mask, Tracheal tube, Patient simulation

## Abstract

**Background:**

Supraglottic devices are helpful for inexperienced providers who perform ventilation in emergency situations. Most supraglottic devices do not allow secondary tracheal intubation through the device. The novel intubating laryngeal tube (iLTS-D®) and the intubating laryngeal mask (Fastrach™) are devices that offer supraglottic ventilation and secondary tracheal intubation.

**Methods:**

We evaluated the novel iLTS-D and compared it to the established Fastrach using a manikin-based study. Participants used both devices in a randomised order. The participants conducted four consecutive trials on a manikin. One trial was composed of the following procedures. First, participants ventilated the manikin using either iLTS-D or Fastrach. ‘Time to ventilation’, success rates and number of attempts were recorded for the supraglottic device. Second, participants intubated the manikin through the previously inserted supraglottic device. ‘Time to tracheal ventilation’, success rate and tube localisation were recorded. The primary endpoint was the results of the final fourth trial, which mirrored the standardised training of trials 1, 2 and 3.

**Results:**

A total of 64 participants were enrolled. All of the participants successfully inserted both devices on their first attempt in trial 4. Fastrach was applied 1 s faster in trial 4 than the iLTS-D (median ‘time to ventilation’ Fastrach: 13.5 s., iLTS-D: 14.5 s., p = 0.04). All participants successfully intubated through both devices in trial 4. There was no difference in ‘time to tracheal ventilation’ by tracheal intubation between either device (median ‘time to tracheal ventilation’: Fastrach: 14.0 s., iLTS-D: 14.0 s., p = 0.16).

**Conclusion:**

The iLTS-D performed similarly to the ILMA in insertion and intubation times in a manikin setting.

## Background

Placement of a tracheal tube requires a high skill level, continuous training and frequent practice [1]. There are several recommendations for coping strategies when tracheal intubation cannot be successfully completed [2, 3]. Supraglottic airway devices are a first-line alternative to tracheal intubation, and they are particularly useful for health care providers who are not trained in tracheal intubation to establish ventilation in emergency patients. The airway is defined as secure for cardiopulmonary resuscitation (CPR) after the proper application of a supraglottic airway device, such as a laryngeal tube or laryngeal mask [4]. However, recent reports indicate that initial endotracheal intubation is associated with improved patient outcomes after cardiac arrest and CPR [5]. Supraglottic devices and tracheal intubation surpass bag-mask ventilation in ventilation and the protection of aspiration during CPR. Consequently, every paramedic- and physician-staffed ambulance in the federal state of Rhineland-Palatinate in Germany is equipped with a set of laryngeal tubes and Macintosh laryngoscopes. However, secondary intubation through a placed laryngeal tube is technically challenging, and it requires additional equipment, such as a fibrescope and an Aintree® Catheter, once the patient reaches the hospital.

The intubating laryngeal mask, Fastrach™ (Teleflex, Buckinghamshire, UK), is a supraglottic airway device that offers the opportunity for secondary tracheal intubation. This device was extensively evaluated in simulated environments [6] and patient care [7, 8]. The Fastrach is generally accepted as an easily learned device, especially for novices, that allows for tracheal intubation with a high success rate [9].

The novel intubating laryngeal tube iLTS-D® (intubating laryngeal tube suction – disposable, VBM Medical Inc., Sulz, Germany) combines the characteristics of laryngeal tube suction [10] (LTS-D®, VBM Medical Inc., Sulz, Germany) with the added possibility of secondary tracheal intubation.

The Fastrach is a well evaluated device. Therefore, we compared the novel iLTS-D to the Fastrach in a manikin model.

We hypothesized that there is no differences in ‘time to ventilation’ (definition: see below) and the success rates of ventilation and intubation between the Fastrach and the novel iLTS-D.

## Methods

The ethics board approved the study (State Physicians’ Chamber of Rhineland-Palatinate, Registration Nr.: 837.336.13 (9021)). We designed a prospective randomised non-blinded observational manikin-based study.

### iLTS-D

**Fig. 1:**
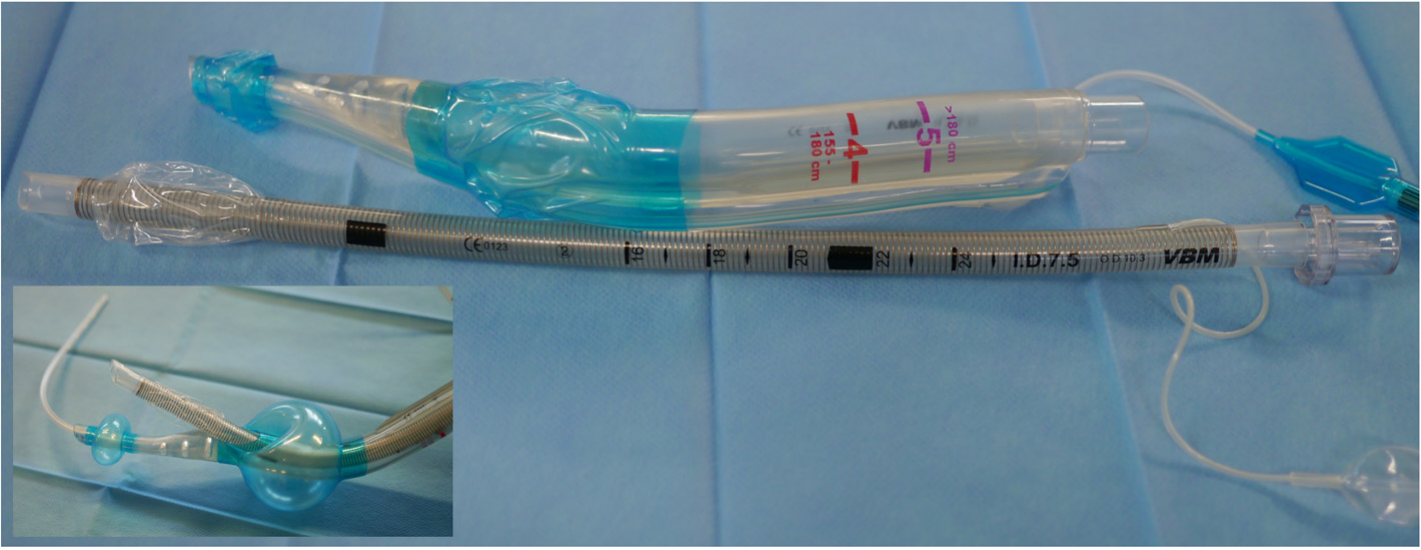
The intubating laryngeal tube suction “iLTS-D”

The iLTS-D (Fig. 1) has been recently introduced and is an advancement of the LTS-D. The insertion technique is similar to the LTS-D. Adequate placement of the device positions the distal end of the ventilation canal at the cranial aspect of the glottis. The opening is shaped as a ‘ramp’ within the canal to guide the tracheal tube in a steep angle in the direction of the glottis. Two half-moon formed wings covering the air canal are meant to lift up the epiglottis when the tube passes. The cuffs of the iLTS-D are configured similarly to the LTS-D. The proximal larger cuff is designed to seal the pharynx cranially of the epiglottis, and the distal smaller cuff is designed to seal the oesophagus at the level of the cricoid cartilage. The iLTS-D is currently available in an adult size, which fits classical LTS-D sizes 4 and 5 simultaneously, with two separate marks that are positioned at the upper front teeth to define the depth of insertion (Fig. 1). The proximal entrance for a gastric tube is shaped like a small cone to ease insertion. The manufacturer provides a specific metal wire armoured cuffed tube with a soft tip and a specially matched “pusher” to allow for the removal of the iLTS-D while the tracheal tube is kept in place.

### Manikin

Manikins are a useful training and evaluation instrument in medical education [11]. However, results of manikin-based studies are difficult to directly transfer to real life situations [12]. Nevertheless, manikins provide a good level of clinical reproducibility of the conditions for supraglottic airway devices, especially for novices [13]. As a successor of the ‘Airway Trainer’ (Laerdal Medical AS, Stavanger, Norway) [14], the ‘ALS Simulator advanced’ manikin (Laerdal Medical AS, Stavanger, Norway) offers sufficiently realistic conditions for the insertion of supraglottic airway devices and the possible application of a tracheal tube.

### Participants

Final year medical students were chosen as participants because this population has good theoretical knowledge but lacks practical experience in airway management. Therefore, these students are a representative sample of health care providers who are recommended to use supraglottic airway devices in emergency situations.

A total of 64 final year medical students were invited, and all invited students participated in the study (22 males (34.4 %) and 44 females (65.6 %)). Written informed consent was obtained. A standardised introduction was given to a maximum of 5 students for this study. The introduction was composed of a demonstration of the application of the Fastrach and the iLTS-D in a manikin and the subsequent intubation through the device. Students were randomly assigned to begin the evaluation with the iLTS-D or Fastrach after the introduction.

### Data collection

The following measurements were included in this study: ‘time to ventilation’ using the supraglottic airway device, number of attempts and success rate of ventilation, ‘time to tracheal ventilation’ using a tracheal tube, localisation of the tracheal tube and success rate of ventilation using the tracheal tube.

A ‘trial’ was defined as follows: A stopwatch was started as soon as the student touched the iLTS-D or Fastrach, and it was stopped when the manikin showed the first ‘normal chest rise’ (‘time to ventilation’) according to ERC guidelines [4]. Therefore, students had to ventilate the manikin immediately after insertion and cuff inflation of the supraglottic device. We used a self-inflating bag because it is a commonly used device for emergency ventilation. A ‘normal chest rise’ was defined as adequate ventilation. The ventilation was counted as a failure if an audible leak occurred with minimal chest rise. Ventilation was counted as successful if an audible leak was present, and a normal chest rise was present. An insertion ‘attempt’ was defined as the procedure of the insertion of a particular device. If the participant took the devices out of the manikin before trying to ventilate and restarted within one trial, it was counted as a second attempt. A third or fourth attempt was defined equivalently. If the participant could not perform ventilation within 60 s after the stopwatch was started, the attempt was defined as unsuccessful [15, 16]. After establishing supraglottic ventilation, ‘time to tracheal ventilation’ using the tracheal tube was measured with the properly inserted supraglottic airway devices in place. The stopwatch was started when the student touched the tracheal tube and stopped when the first normal chest rise was detected (‘time to tracheal ventilation’). A time limit of 60 s was used.

This trial was repeated four times, defined as trial 1 to trial 4, for the supraglottic device and trial ‘tracheal’ 1 to 4 (trial T 1 – 4) for intubation through the particular device. Each student was asked to grade each device from 1 (best) to 6 (worst) after four trials with one device. The participant proceeded to the other device after completing 4 trials with one device.

### Study outcomes

The primary endpoint was the difference in ‘time to ventilation’ after trial 4 between the Fastrach and iLTS-D. We chose trial 4 as the primary endpoint to allow the volunteers a training of exactly three applications to standardise their experience with the devices.

Secondary endpoints were the differences in ‘time to ventilation’ using the supraglottic device in trials 1 to 3, differences in ‘time to tracheal ventilation’ through the particular device in trials T 1 to T 4, differences in ‘time to ventilation’ between trial 1 and trial 4, and trial T 1 and trial T 4 for the particular supraglottic device, which mirrored the training effect and practice. Further secondary endpoints were differences in success rates, the ‘number of attempts’ required to properly apply the supraglottic device, and the localisation of the tracheal tube.

### Sample size and statistical analysis

Analysis was conducted using IBM SPSS Statistics Version 22 (IBM, Ehningen, Germany) SAS Version 9.4 (SAS Institute Inc., Cary, USA) and Microsoft Excel 2010 (Microsoft, Redmond, USA). Data are displayed as medians, minimums, maximums and interquartile range using boxplot. Differences were examined using the Wilcoxon-Rank-Test. The statistical significance level was set at α < 0.05.

Inexperienced participants in a previous study needed an average of 20 s to ventilate using the Fastrach (standard deviation (SD): 14 s) [6]. A relevant improvement in ‘time to ventilation’ in trial 4 would be a 7-s reduction in the average time to ventilation (i.e., 0.5 SD). We assumed that there was a small positive correlation (r = 0.1 to 0.3) between times to ventilation using the Fastrach and iLTS-D. A normal distribution of times to ventilation was assumed, and an average difference of 7 s can be established in a two-sided signed rank test at the 5 % level with a power between 83.3 % for r = 0.1 and 91.2 % for r = 0.3 with the inclusion of 64 subjects. A greater positive correlation would allow the establishing of an average difference of 7 s with an even higher power.

## Results

The 64 participants performed a total of 256 attempts for each device over all four trials. All attempts were completed within the time limit of 60 s. We displayed primary and secondary endpoints separately for statistical accuracy.

### Supraglottic Airway Management: ventilation after trial 4

**Fig. 2:**
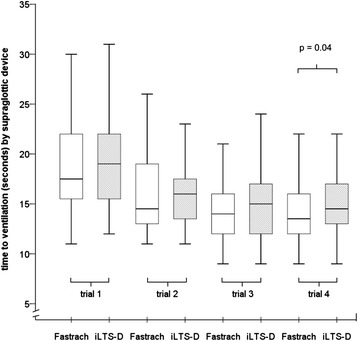
“Time to ventilation” in seconds using the Fastrach (FT) and iLTS-D

Ventilation was established 1 s faster using the Fastrach than with the iLTS-D in trial 4 (median ‘time to ventilation’ trial 4: Fastrach: 13.5 s., [IQR 12.0 – 16.0 s.], iLTS-D: 14.5 s. [13.0 – 17.0 s.], p = 0.04) (Fig. 2, Table 1).

**Table 1:** “Time to ventilation” in seconds using the supraglottic devices

trial	1		2		3		4	
device	**Fastrach**	**iLTS-D**	**Fastrach**	**iLTS-D**	**Fastrach**	**iLTS-D**	**Fastrach**	**iLTS-D**
median	17.5	19.0	14.5	16.0	14.0	15.0	13.5	14.5
minimum	11	12	11	11	9	9	9	9
maximum	34	60	48	29	25	24	22	30
IQR	15.25-22.0	15.25-22.0	13.0-19.0	13.25-17.75	12.0-16.0	12.0-17.0	12.0-16.0	13.0-17.0
p-value	0.57		0.71		0.19		0.04	

Time points: consecutive trials 1, 2, 3, 4; median, minimum, maximum, upper and lower quartile of ‘time to ventilation’ for the particular instrument displayed as table and boxplot; after trials 1, 2 and 3, the Fastrach could be inserted 1 s faster than the iLTS-D in trial 4 (primary endpoint: Fastrach: median: 13.5 s., iLTS-D: 14.5 s., p = 0.04). Though statistically significant, 1 s is not considered to be a relevant amount of time

### Supraglottic Airway Management: secondary endpoints

There were no differences in ‘time to ventilation’ between the Fastrach and the iLTS-D in trials 1 to 3 (Fig. 2, Table 1).

All students successfully used the Fastrach in their first attempt in trial 1. One student (1.6 %) needed two attempts using the iLTS-D to achieve successful ventilation. All students were successful using both devices on the first attempt in trials 2, 3 and 4.

The training effect over all four trials yielded a benefit of a 4-s faster ‘time to ventilation’ from trials 1 to 4 using the Fastrach (median ‘time to ventilation’: FT trial 1: 17.5 s., trial 4: 13.5 s., p < 0.001), and a 4.5-s faster time using the iLTS-D (median ‘time to ventilation’: iLTS-D: trial 1: 19.0 s., trial 4: 14.5 s., p < 0.001) (Fig. 2).

### Tracheal intubation trough device

There was no difference in ‘time to ventilation’ through a tracheal tube between the Fastrach and iLTS-D in trial T 1. Trials T 2 and T 3 showed faster ventilation times using the Fastrach. No differences between devices were observed in trial T 4 (Table 2).

**Table 2:** ‘Time to tracheal ventilation’ in seconds using the tracheal tube

trial	T 1		T 2		T 3		T 4	
device	**Fastrach**	**iLTS-D**	**Fastrach**	**iLTS-D**	**Fastrach**	**iLTS-D**	**Fastrach**	**iLTS-D**
median	19.0	19.0	15.0	16.0	14.0	15.0	14.0	14.0
minimum	9	10	10	9	10	10	9	9
maximum	52	60	29	60	33	60	50	42
IQR	16.0-24.75	16.0-22.75	12.25-17.0	14.0-19.0	12.0-16.0	13.0-17.75	12.0-16.5	12.0-17.0
p-value	0.72		0.03		0.04		0.16	

Time points: consecutive trials 1, 2, 3, 4; median, minimum, maximum, upper and lower quartile of ‘time to ventilation’ for the particular instrument displayed as boxplot; after trials 1, 2, and 3, there was no difference in ‘time to ventilation’ for the tracheal tube inserted through the Fastrach and the iLTS-D in trial 4 (Fastrach: median: 14.0 s., iLTS-D: 14.0 s., p = 0.16)

Intubation via the Fastrach was successfully on the first attempt in 98.4 % of the attempts in trial T 1 (63/64). All attempts were successful on the first attempt in trials T 2, 3 and 4. The success rate on first attempt was 98.4 % (63/64) using the iLTS-D in trials T 1, 2 and 3. All participants placed the tube correctly on the first attempt in trial T 4.

Both devices received comparable grades by participants (Fastrach: 2, IQR: 1–2; ILTS-D: 1.5 IQR: 1–2; p = 0.87).

## Discussion

This study demonstrated that the intubating laryngeal mask Fastrach was applied 1 s faster after four trials of insertion attempts compared to the novel iLTS-D in a manikin. Ventilation was established within 13.5 s using the Fastrach and within 14.5 s using the iLTS-D. However, these differences are unlikely to be clinically relevant.

‘Time to ventilation’ showed a favourable tendency of faster application after four trials. Tracheal intubation through the Fastrach was performed as fast as the iLTS-D after four trials (both devices: 14 s).

Both instruments achieved success rates of over 98 %, and ventilation was achieved within one minute under simulated conditions. ‘Time to tracheal ventilation’ revealed no differences between instruments after four trials. Therefore, the iLTS-D is a feasible device for airway management under simulated conditions.

Manikin-based studies are not readily comparable because of the use of a wide variety of methods, assessment tools and analyses. Furthermore, data collected on manikins hardly transfer to real patients [17]. Possibly harmful overestimations of the clinical capability in inexperienced subjects after simulator training were reported [18]. The simulated anatomy of the airway has developed to an acceptable level of realism [14], but educators recommend the use of various airway models to achieve adequate training for airway management novices [19]. Anatomical distances between the upper front teeth and the glottis are not always adequately represented in many manikins. These landmarks are important for the insertion depth of airway devices and could potentially result in an easier positioning of tubes and supraglottic devices. Evaluation of airway instruments in manikins does not replace clinical evaluation. The first experiences of a novel device in a manikin setting represent the first step to fully understanding the device before it is applied in human patients. The authors believe that a device that fails in manikins should not be used directly in human patients.

These factors must be taken into account when discussing the clinical implications. LTS-D is commonly used in pre-hospital emergency medicine mostly in Germany, United States of America and Japan. Paramedics are well trained in the handling of laryngeal tubes in pre-hospital care, and they use these devices as a primary tool for airway management or in cases of failed intubation [20]. The use of supraglottic devices is generally accepted as an important strategy to maintain oxygenation, especially in failed endotracheal intubation situations. However, most supraglottic devices only allow for ventilation. Additional devices (e.g., flexible fibrescope with Aintree catheter) are necessary for secondary intubation through the device, and tube placement can be challenging. The exchange of the supraglottic device for a tracheal tube may be challenging, and it carries the risk of hypoxia in patients with present airway trauma or difficult anatomy. An increasing number of reports show secondary swelling of the airway when supraglottic devices were placed in emergency situations, which is likely because of tissue injury due to uncontrolled cuff pressure [21]. The iLTS-D may be a promising supraglottic airway device that bridges the gap between supraglottic airway management and tracheal intubation, especially in difficult airway situations because the tube can be placed through the device without removal of the iLTS-D. Paramedics or inexperienced providers could use the device for ventilation in the pre-hospital setting, and an endotracheal tube can be placed after hospital admittance. The iLTS-D may be used as a primary device for tracheal intubation, which could affect cardiac arrest survival, if the first reports that showed improved CPR outcomes with the application of tracheal intubation can be verified.

Studies of the Fastrach show comparable success rates to the present study. Ventilation was achieved within 1 min in a simulated setting [22]. The Fastrach was successfully placed within 1 min even while personnel was wearing chemical, biological, radiation, or nuclear-personal protective gear [23].

A study of 119 inexperienced students demonstrated that tracheal intubation could be better achieved using the Fastrach than the classical laryngoscope [15]. None of the subjects failed to perform tracheal intubation using the Fastrach. Success rates of 90 % in the first attempt, 8 % in the second and 2 % in the third attempt within 60 s were described.

Four airway devices (Macintosh blade, McCoy, Airtraq, Fastrach) were compared in a manikin study (SimMan®, Laerdal, Stavanger, Norway) [24]. The median time to intubation using the Fastrach was 21.6 s in a normally adjusted airway. These data are consistent with the results of this study.

Currently there are no published data on the iLTS-D. However, the iLTS-D design is very similar to the widely used LTS-D. A simulation-based study evaluated six airway devices (classical laryngeal mask, Fastrach, LMA ProSeal, Laryngeal tube, Combitube, conventional tracheal tube) in manikins using health care professionals. In contrast to the present study, ‘time to ventilation’ was prolonged (55.2 to 69.5 s). However, Fastrach was only used as a conduit for intubation. Using the laryngeal tube, time to insertion was 10.8 to 13.0 s in the 1^st^ attempt and 9.5 to 10.4 s in the 3^rd^ attempt. Our results showed a prolonged insertion time, which may be due to the training level of the participants included in this study [25].

Further simulation scenario-based studies on manikins yielded similar success rates of over 95 % and time to ventilation of up to15 s for laryngeal tubes [16, 20].

There are other supraglottic airway devices that enable endotracheal intubation and the placement of a gastric tube, such as the laryngeal mask i-gel™ or Air-Q™. However, blind tracheal intubation could not be performed as sufficiently as the Fastrach through these devices [26, 27].

Gastric distension can potentially develop into a serious life-threatening condition. There are some case reports of this problem with supraglottic airway devices, especially in emergency medicine [28, 29]. The iLTS-D features the opportunity of a gastric tube similarly to the LTS-D. A comparison of the LTS-D to the ProSeal laryngeal mask found no difference in the facilitation of gastric tube placement (successful gastric tube insertion: ProSeal: 97 %, LTS-D: 96 %) [30]. This comparison should be further investigated using the iLTS-D.

The results of this study offer an evaluation of a novel supraglottic airway device that tries to bridge the gap to tracheal intubation for in- and out-of-hospital emergency patients.

## Conclusion

The iLTS-D showed good performance on a manikin, which was comparable with the Fastrach in time to ventilation and intubation and insertion success rates. The iLTS-D combines the well-known advantages of laryngeal tube with the possibility of secondary tracheal intubation and gastric access. These results revealed a similar performance of iLTS-D to the Fastrach. This novel device has the potential to improve emergency airway management given the wide distribution of laryngeal tubes in Germany, Japan and the United States of America. However, clinical trials examining the iLTS-D are mandatory before this device is introduced into daily practice.
